# Ex vivo confocal microscopy for surgical margin assessment: A histology‐compared study on 109 specimens

**DOI:** 10.1002/ski2.91

**Published:** 2022-01-10

**Authors:** L. Grizzetti, F. Kuonen

**Affiliations:** ^1^ Department of Dermatology and Venereology, Hôpital de Beaumont Lausanne University Hospital Center Lausanne Switzerland

## Abstract

**Background:**

The assessment of surgical margins is mandatory to prevent local recurrence or distant dissemination of skin cancers. Histological examination of haematoxylin and eosin (H&E)‐stained slides from paraffin‐embedded or frozen samples is the gold standard for margin assessment, but is a time‐consuming procedure. Ex vivo confocal laser scanning microscopy (CLSM) is an upcoming technique that scans unfixed fresh tissue rapidly, allowing fast per‐operative margin assessment.

**Objective:**

Here, we propose to assess the efficiency of a new ex vivo confocal microscope for the per‐operative assessment of surgical margins.

**Methods:**

We analyzed 16 biopsies and 93 surgical specimens of basal cell and squamous cell carcinomas by ex vivo CLSM using Histolog® Scanner V2. Surgical specimens included fusiform excisions, slow‐Mohs peripheral and deep compartments, and Mohs excisions. The time required from surgical excision to image analysis was recorded and the quality of the images obtained for each specimen assessed. The presence or absence of tumour was estimated based on ex vivo CLSM images and compared with conventional H&E‐stained sections from paraffin‐embedded or frozen (Mohs) specimens.

**Results:**

Mean time for specimen processing using Histolog Scanner was 5.1 ± 3.4 min. We obtained 89% of high quality images. Mean time for confocal image analysis was 1 ± 0.76 min. The diagnostic sensitivity and specificity for ex vivo CLSM compared to classical H&E procedures were respectively 93% and 100% when performed on tumour biopsies. The overall sensitivity and specificity for ex vivo CLSM for margin assessment compared to classical H&E procedures were respectively 61.5% and 95%, with variations depending on the type of tumour or surgical specimen analyzed. In particular, we obtained 80% sensitivity and 100% specificity for the assessment of BCC surgical margins.

**Conclusion:**

Our data suggest that ex vivo CLSM using Histolog® Scanner V2 could be a valid help for surgeons for a fast and accurate per‐operative margin analysis.

## Graphical abstract

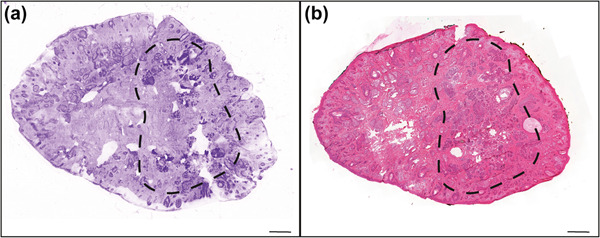

1

**What's already known about this topic?**

- Ex vivo fluorescence confocal laser scanning microscopy (CLSM) allows fast histopathological analysis of fresh tissues
- CLSM has a high sensitivity and specificity for the assessment of basal cell carcinoma surgical margins

**What does this study add?**

- Our study reports the diagnostic accuracy of a new ex vivo confocal microscope
- Furthermore, it compares the sensitivity and specificity of CLSM according to tumour types and surgical specimen

## 2 INTRODUCTION

Basal (BCC) and squamous (SCC) cell carcinoma are the most frequent cancers in human, for which the gold standard therapy is surgical removal with histopathological analysis of the surgical margins to assess complete excision.^1^, ^2^ For high‐risk tumours, three‐dimensional micrographic analysis (Mohs micrographic or slow‐Mohs micrographic techniques) is required to reduce the risk of relapse.^3^ While conventional histopathological analyzes assess 1%–2% of the surgical margins, micrographic analyzes are expensive, time‐consuming and unfortunately not widely available procedures.

In the last years, ex vivo confocal laser scanning microscopy (CLSM) has developed as an optical method allowing high‐resolution images of fresh, unfixed tumour tissue specimens like BCC, SCC, dermatofibroasarcoma and other adnexal tumours.^4^ Ex vivo CLSM allows fast analysis, sparing the time‐consuming and costly procedure of tissue fixation, cutting and staining. In addition, it allows complete analysis of the surgical margins. The diagnostic accuracy of ex vivo CLSM compared to histopathological analyzes has been evaluated in numerous studies, mostly for BCC,^5^, ^6^, ^7^, ^8^, ^9^, ^10^, ^11^ although with varying sensitivity (73%–100%) and specificity (89%–100%) rates. Horn et al reported high sensitivity (95%) and specificity (96%) for SCC too.^12^

**FIGURE 1:**
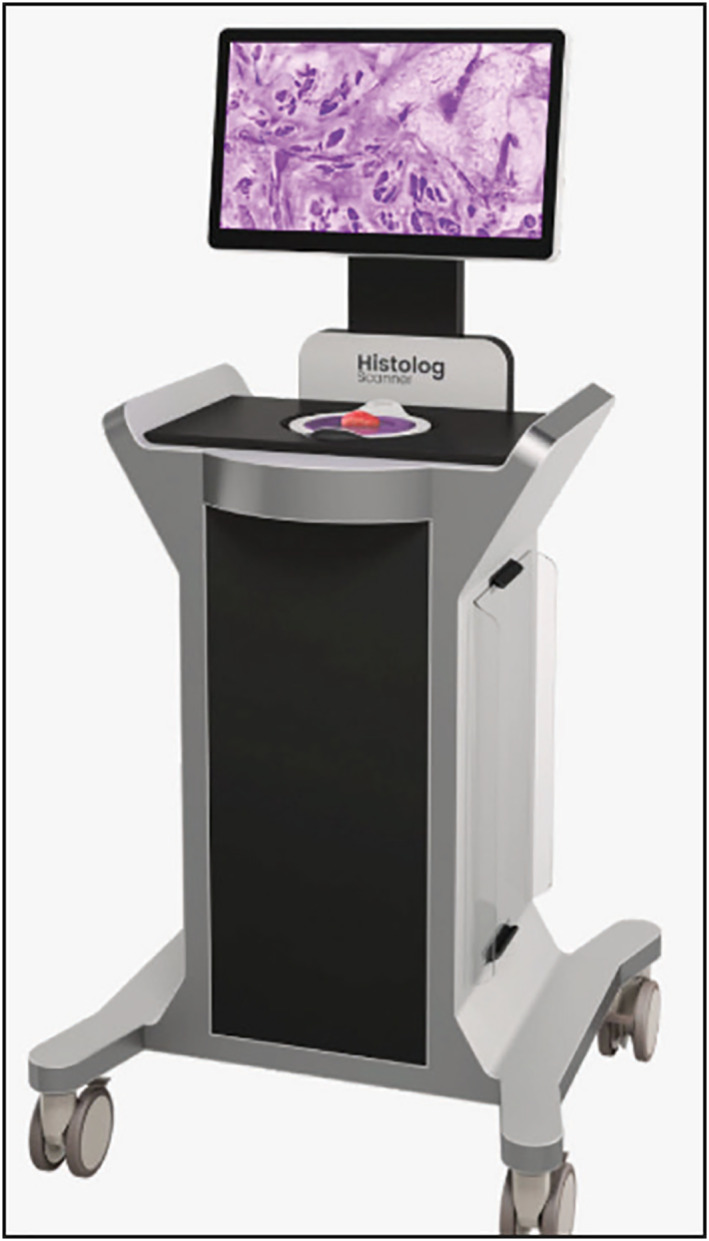
Histolog® Scanner V2

Here, we tested the novel, improved Histolog® Scanner V2 (Figure 1) for the ex vivo assessment of BCC and SCC biopsies and surgical margins in a clinical setting and workflow. To do so, we compared the confocal digital images to H&E‐stained frozen (Mohs) or paraffin‐embedded sections.

## 3 MATERIAL AND METHODS

### 3.1 Setting

Between January and March 2021, we prospectively analyzed 109 specimens, of which 52 BCC (48.7%), 55 SCC (50.4%) and 2 basosquamous carcinomas (BSC; 1.8%) with Histolog® Scanner V2 (Figure 1) at Lausanne University Hospital. The study design was approved by the institutional review board of Lausanne University Hospital CHUV, and the local ethics committee (study 2015‐00187). Each patient enroled provided written informed consent. Inclusion criteria were age >18, clinically diagnosed BCC or SCC, for which surgery was indicated. Exclusion criteria was age <18. Histolog® Scanner V2 is a 488 nm fluorescence‐based confocal laser scanning microscope with 2 µm lateral resolution, approximately 30 µm penetration depth and a 48 × 36 mm field of view, manufactured and provided by Samantree Medical SA, Switzerland. Images of the full field of view can be obtained either in a “preview”, low‐resolution (15 s) or in an “acquisition”, high‐resolution mode (1 min). LG and FK were the dermatosurgeons. LG and FK evaluated the confocal digital images. Before the start of the study, they both had a full‐day instruction on how to use the Histolog® Scanner V2. Slow‐Mohs and standard paraffin‐embedded sections were evaluated by an independent dermatopathologist. FK is the Mohs surgeon who evaluated the H&E staining of Mohs frozen sections. The different types of tumours are shown in Table 1. Mean age of patients was 74.1 years old, with a range between 39 and 98 years. Percentage of male and female patients was 76.9% and 23.1% respectively. All BCC and SCC subtypes (including 2 BSC) were included.

**TABLE 1:** Frequency of different tumour type specimens

Tumour type	Subtype	Number	%
BCC		52	47.7
	Nodular	18	16.5
	Micronodular	9	8.3
	Infiltrative	22	20.2
	Superficial	3	2.8
SCC		55	50.5
	Well differentiated	22	20.2
	Moderately differentiated	20	18.3
	Poorly differentiated	8	7.3
	In situ	5	4.6
Baso‐squamous carcinoma		2	1.8

### 3.2 Procedure

Before imaging on CLSM, slow‐Mohs specimens were excised with a 2–3 mm margin, perpendicular to skin surface incision and prepared according to the “Tuebinger Torte” method, creating bases (deep margin) and lateral samples (peripheral margins).^13^ Deep margins of fusiform excisions were imaged without prior tissue dissection. Mohs samples were excised with a 45° angles. Debulking and flattening were performed as required for standard preparation for Mohs micrographic analysis. Altogether, we distinguished biopsies and surgical margin specimens, including: deep margins (from fusiform excisions and slow‐Mohs bases), peripheral margins (slow‐Mohs lateral surgical margins) and Mohs excisions. Importantly, obtaining adequate positioning of the specimen on the Histolog® dish, in order to get high quality images, was the most time‐consuming part of the whole procedure.

Every fresh specimen was stained for 10 s with a fluorochromatic dye which binds to nucleic acids and negatively charged proteins (Histolog Dip®), and subsequently rinsed in a phosphate buffer solution. The specimen was then placed on a special transparent dish (Histolog® Dish) and applied on the scanning system for laser analysis. Except for a very large specimen (10 × 6 cm), the imaging area (48 × 36 mm) allowed full viewing of the specimens in a single window. As Histolog® Scanner V2 only detects a single plane, it was essential to bring the area to scan exactly into contact with the surface of the dish. Therefore, in addition to tissue dissection (when necessary), further flattening was achieved by exerting pressure on the specimen (using a flour‐filled vinyl glove, e.g.). Fast (15 s), low‐quality “preview” imaging was used for immediate evaluation of optimal specimen positioning and flattening. Once obtained, a longer (1 min), high‐quality image was taken for subsequent analysis. Regularly, surgical specimens had to be re‐positioned for optimal viewing of different parts of the specimen in case of irregular flattening for example. Of note, suboptimal resolution was observed in case of insufficient flattening of the specimen or too much pressure exerted on the specimen. The images were then anonymized and saved for later analysis. After image creation, the specimen was fixed in 4% formaldehyde for standard histology processing or frozen and OCT‐embedded for per‐operative Mohs analysis.

Confocal digital images were analyzed using Histolog® software on Histolog® Scanner V2 (to mimick immediate per‐operative assessment), by LG and FK with no prior preparation. For confocal analysis, BCC‐specific criteria included demarcated fluorescent areas with higher nuclear density, peripheral palisading, clefting and nuclear polymorphism.^14^ For SCC, confocal criteria included “black” sharply demarcated irregular areas in the epidermis and dermis (erosion/ulceration), disarray of the normal architecture of the skin, irregular aggregates of cells that are larger than inflammatory cells, keratin pearls and peritumoral inflammatory infiltrate.^15^, ^16^ To facilitate digital image analysis, LG and FK used the zoom, contrast and black&white versus purple (H&E‐like) digital modes allowed by the Histolog® software. Every confocal image was analyzed by two examiners and scored according to quality (very low, low, high, very high quality), based on the following criteria: the displaying of the whole epidermidis (for peripheral margins); the absence of “air bubbles”; the absence of blurred areas (due to haemorrhage or to movement‐induced stitched mosaic artefacts) or areas of lower resolution (likely caused by suboptimal contact of the specimen with the dish, in case of insufficient flattening or too much pressure exerted on the specimen). Very high, high, low and very low quality were attributed according to the proportion (<5%, 5‐10%, 10‐30% and >30% respectively) of the analyzed area harbouring the above‐mentioned criteria. The times required to obtain (including staining and flattening process) and analyze the definitive images were registered. Confocal diagnoses were compared to conventional histopathological H&E analyzes. The performance of ex vivo CLSM was evaluated by calculating sensitivity, specificity, positive and negative predictive values, both globally, and according to tumour or surgical specimen types.

## 4 RESULTS

**FIGURE 2:**
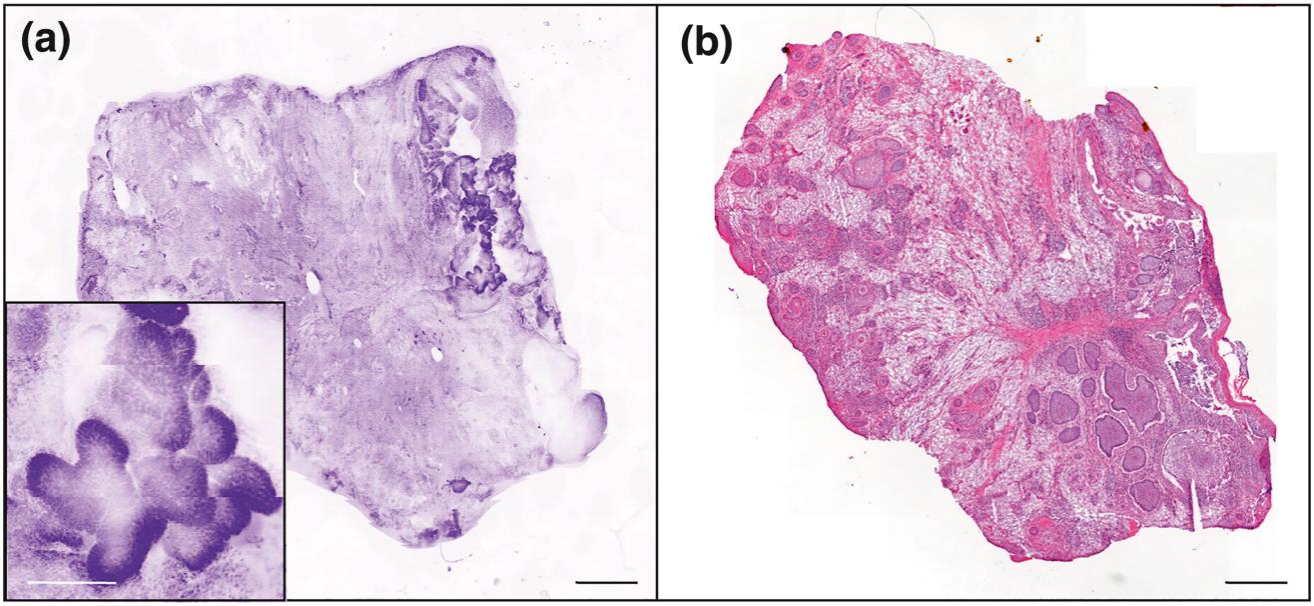
Confocal laser scanning microscopy (CLSM) image (a) and corresponding H&E staining (b) of a basal cell carcinoma (BCC). Scale bars indicate 1000 µm in the bigger panels and 200 µm in the smaller panel

**FIGURE 3:**
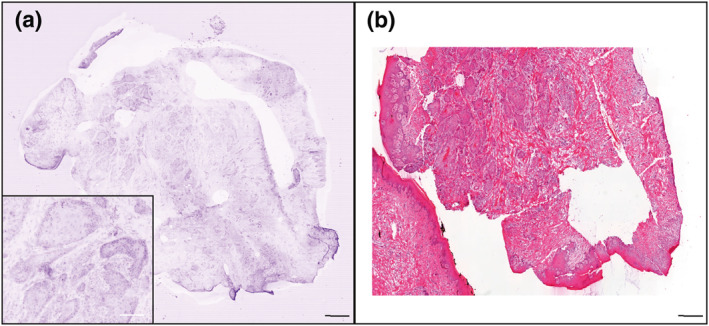
Confocal laser scanning microscopy (CLSM) image (a) and corresponding H&E staining (b) of a squamous cell carcinoma (SCC). Scale bars indicate 1000 µm in the bigger panels and 200 µm in the smaller panel

**FIGURE 4:**
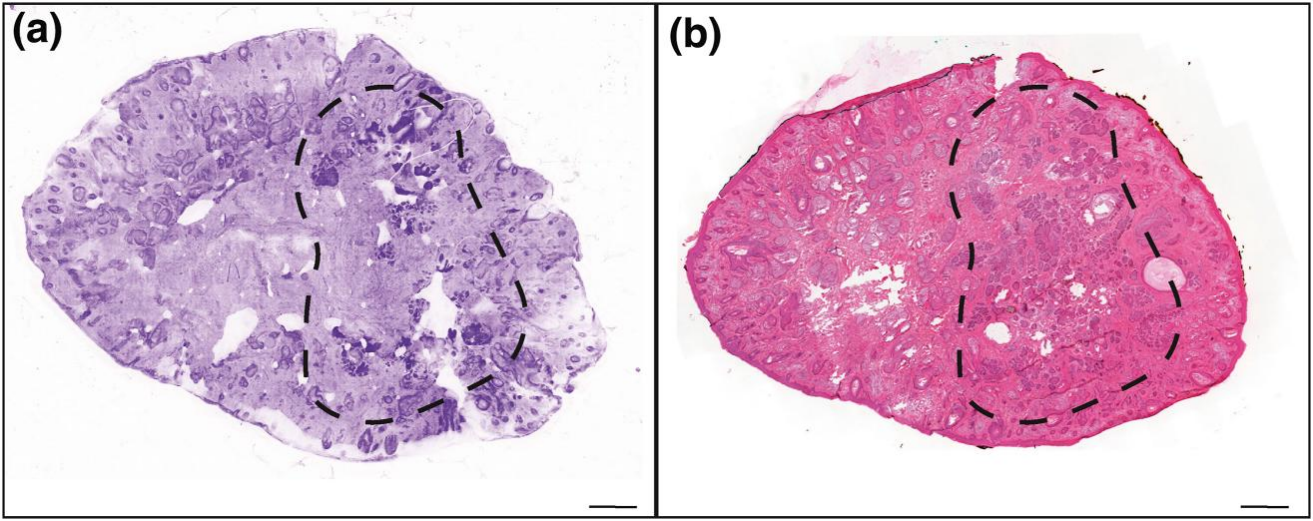
Confocal laser scanning microscopy (CLSM) image (a) and corresponding H&E staining (b) of a Mohs surgical specimen. Dotted circles indicate residual BCC foci. Scale bars indicate 1000 µm

The mean time for specimen processing (including flattening) and acquisition of the image was 5.1 ± 3.4 min (range: 2–15 min). We obtained 89% (*n* = 97) high and very high quality images, with 61% (*n* = 67) of very high quality images (Table 2). Both BCC and SCC confocal features were recognized (Figures 2, 3, 4). The mean time for confocal image analysis was 1 ± 0.76 min (range: 30 s–5 min). The diagnostic sensitivity and specificity of ex vivo CLSM compared to classical H&E procedures were respectively 93% (95% CI: 66.1%–99.8%) and 100% (95% CI: 15.8%–100%) when assessed on tumour biopsies. Consistently with previous reports, we found higher sensitivity for BCC (100%; 95% CI: 66.4%–100%) than for SCC (80%; 95% CI: 28.4%–99.4%; Table 3). Calculated positive and negative predictive values were both 100% for BCC biopsies, while 100% and 50% respectively for SCC biopsies.

**TABLE 2:** Quality assessment of confocal digital images

Image quality	Number	%
Very high	67	61.5
High	30	27.5
Low	8	7.3
Very low	4	3.7

**TABLE 3:** Sensitivity and specificity of ex vivo CM using Histolog Scanner compared to histologic analysis on paraffin‐embedded or frozen (Mohs) H&E sections

	n	True positive	False positive	True negative	False negative	Sensitivity (%)	Specificity (%)
Biopsies	16	13	0	2	1	93 (CI: 66.1%–99.8%)	100 (CI: 15.8%–100%)
Tumour type							
BCC	10	9	0	1	0	100 (CI: 66.4%–100%)	100 (CI: 2.5%–100%)
SCC	6	4	0	1	1	80 (CI: 28.4%–99.4%)	100 (CI: 2.5%–100%)
Surgical margins	93	8	4	76	5	61.5 (CI: 31.6–86.1)	95 (CI: 87.7–98.6)
Tumour type							
BCC	42	4	0	37	1	80 (CI: 28.4–99.4)	100 (CI: 90.6–100)
SCC (including BSC)	51	4	4	39	4	50 (CI: 15.7–84.3)	91 (CI: 77.9–97.4)
Surgical specimen type							
Depth	39	1	2	35	1	50 (CI: 1.3–98.8)	95 (CI: 81.8–99.3)
Periphery	30	2	2	24	2	50 (CI: 6.8–93.2)	92 (CI: 74.9–99)
Mohs	24	5	0	17	2	71 (CI: 29–96.3)	100 (CI: 80.5–100)

Abbreviation: CI, 95% confidence intervals.

The global sensitivity and specificity for surgical margin assessment using ex vivo CLSM compared to classical H&E procedure were respectively 61.5% (95% CI: 31.6%–86.1%) and 95% (95% CI: 87.7%–98.6%; Table 3). Again, we found higher sensitivity (80%; 95% CI: 28.4%–99.4%) and specificity (100%; CI: 90.6%–100%) for BCC than for SCC (50%; 95% CI: 15.7%–84.3%, and 91%; 95% CI: 77.9%–97.4%, respectively; Table 3). Calculated positive and negative predictive values for surgical margins assessment were 100% and 97% for BCC, while 50% and 90.7% for SCC. Regarding the different types of surgical margins, sensitivity and specificity were respectively 50% (95% CI: 1.3%–98.7%) and 95% (95% CI: 81.8%–99.3%) for deep margins (fusiform excision and slow‐Mohs bases compartment), 50% (95% CI: 6.7%–93.2%) and 92% (95% CI: 74.9%–99%) for peripheral margins (slow‐Mohs lateral compartments), and 71% (95% CI: 29%–96.3%) and 100% (95% CI: 80.5%–100%) for Mohs excisions (Table 3). Calculated positive and negative predictive values were 33.3% and 97.2% for deep margins, 50% and 92.3% for peripheral margins and 100% and 89.5% for Mohs specimens.

## 5 DISCUSSION

Here, we report the sensitivity and specificity for surgical margin assessment of skin cancers using the improved version of the CLSM Histolog® Scanner (V2). Overall, our study reports high quality images in most cases (89%) after adequate flattening and positioning of the tissue. Stitched mosaics previously reported with other microscopes^14^ were rarely observed. Globally, we found similar sensitivity and specificity in surgical margin assessment when compared to conventional H&E histopathological analyzes than previous reports performed with different microscopes.^4^ In particular, despite the limited penetration depth (approximately 30 µm), we report similar sensitivity (80% vs. 79.8%–99%) and specificity (100% vs. 80%–100%) for BCC detection compared to VivaScope2500,^7^, ^9^, ^10^ while lower sensitivity (50% vs. 95%) and specificity (91% vs. 96%) for SCC detection.^12^ Thus, given its larger field of view, its accessibility for up to 10 cm‐size specimens, and its faster acquisition times (“preview” 15 s, “acquisition” 1 min), Histolog® Scanner V2 appears particularly suitable for the surgical margins assessment of large BCC specimens.

**FIGURE 5:**
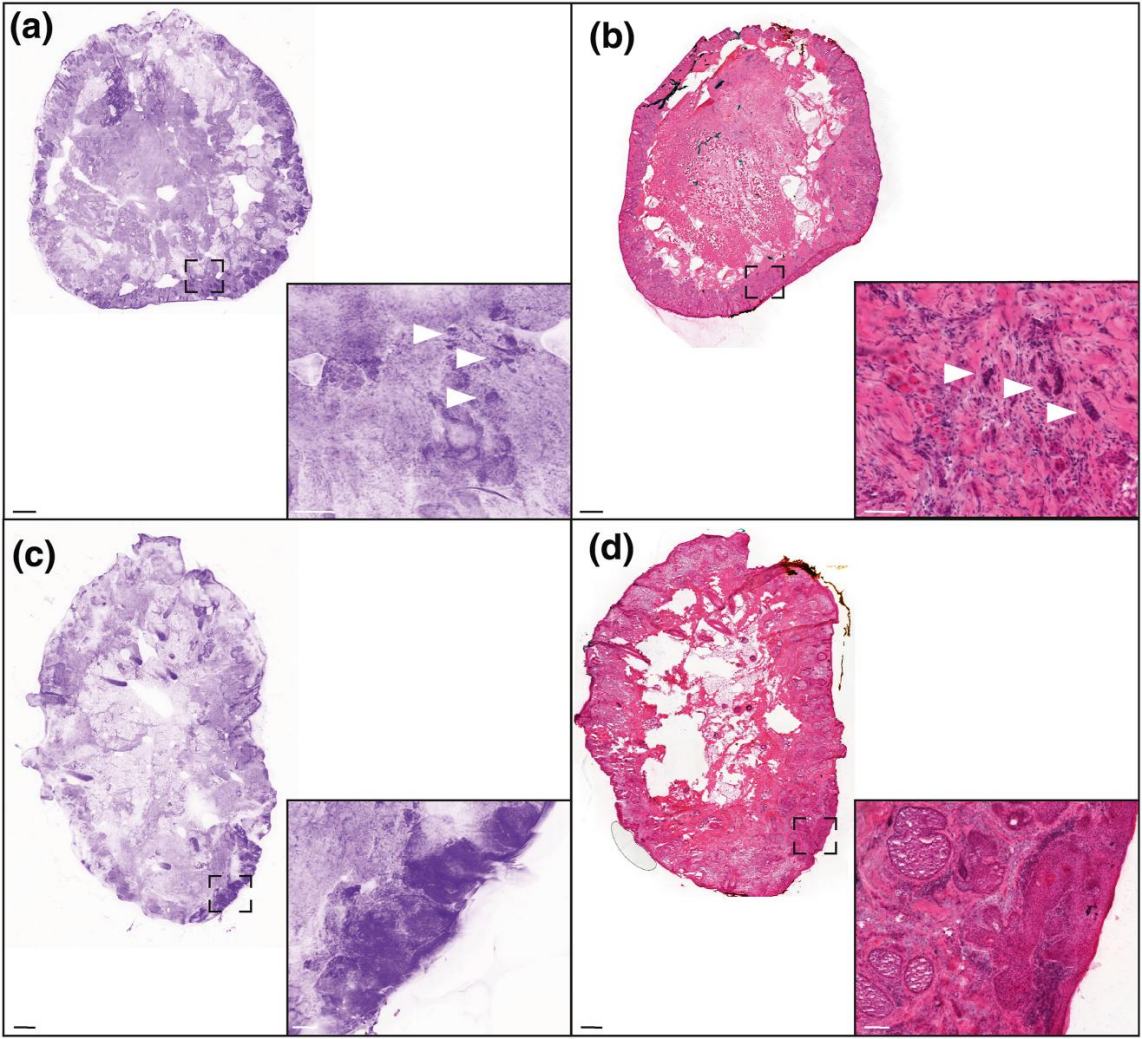
Confocal laser scanning microscopy (CLSM) images (a,c) and corresponding H&E stainings (b,d) of Mohs surgical specimens. (a) and (b) illustrate a false‐negative CLSM picture of a highly infiltrating BCC. Arrowheads indicate tumour cell clusters on H&E (b) that were missed on the CLSM picture (a). (c) and (d) illustrate a false‐negative CLSM picture of a residual peripheral in situ SCC. Dotted lines indicate the magnified areas. Scale bars indicate 1000 µm in the bigger panels and 200 µm in the smaller panels

When looking specifically into BCC surgical margins, we report excellent positive and negative predictive values (100% and 97% respectively), further establishing the potential role of ex vivo CLSM for BCC surgical margin assessment in the clinics. Importantly, the false negative case was a highly infiltrative (sclerodermiform) BCC composed of very thin tumours islands, which were missed on the confocal digital image (Figure 5, *upper panels*). Previous data report the detection by ex vivo CLSM of very thin infiltrative BCC tumour islands in perineural or perivascular invasion,^17^ using however other confocal microscopes combining reflectance and fluorescence mode with possible deep scanning into the specimen. This example illustrates the limited resolution offered by the Histolog® Scanner V2, and the difficulty of recognizing highly infiltrative BCC foci. Although encouraging, the analysis of SCC specimens highlighted the lower positive and negative predictive values (50.0% and 90.7% respectively) for margin assessment using ex vivo CLSM, reflecting its more difficult histopathological analysis.

**FIGURE 6:**
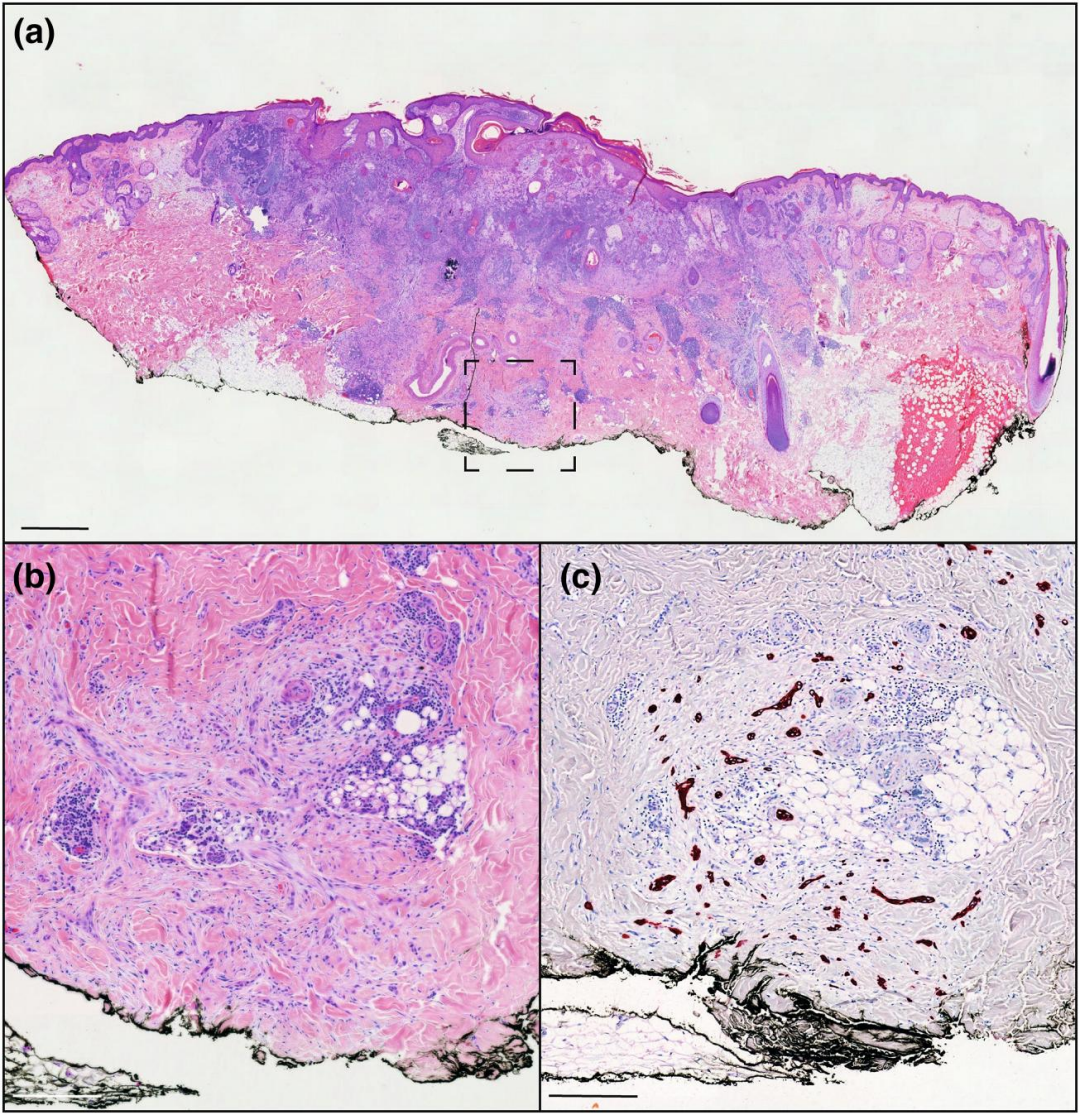
H&E stainings (a,b) and pan‐cytokeratin immunostaining (c) showing small infiltrating tumour cell clusters on the deep margin of a basosquamous carcinoma (BSC) excision sample. Dotted lines indicate the magnified area shown in (b) and (c). Scale bar in (a) indicates 1000 µm. Scale bars in (b) and (c) indicate 200 µm

Our study also reports the specific diagnostic accuracy of ex vivo CSLM for biopsies, deep and peripheral margins or Mohs specimens. While we found high diagnostic sensitivity (93%; 95% CI: 66.8%–99.3%) and specificity (100%; 95% CI: 15.8%–100%) for biopsies, the negative predictive value was relatively low (66.7%), mostly due to the low number of tumour‐free and the difficult positioning/flattening of thin, firm biopsy samples, hence lower image quality. Deep and peripheral margins assessment revealed low diagnostic accuracy with 33.3% and 50% positive predictive value respectively, but higher negative predictive values (97.2% and 92.3% respectively). In particular, the one false negative deep margin resulted from a highly infiltrative basosquamous carcinoma (Figure 6). At this point, it is unclear whether this resulted from the limited detection by Histolog® Scanner V2 of very small tumour cell clusters, or from a histological “false positive” as no tumour island was found in the 30 µm deepest margin, analyzed by CLSM. Of note, the “false negative” cases observed in the peripheral margins assessment, may actually represent histological “false positive” from tissue loss during paraffin‐embedded tissue sectioning during conventional histopathological procedure, as previously reported.^11^ In the contrary, the “false positive” observed in the deep margin assessment using ex vivo CLSM may actually represent histological “false negative”, due to the limited (1%–2%) margin assessment of deep margins during conventional H&E examining.^3^ Remarkably, the assessment of Mohs specimens revealed higher sensitivity (71%) and specificity (100%) compared to deep and peripheral margin assessment, with positive and negative predictive values of 100% and 89.5% respectively. In favour of higher diagnostic accuracy are the debulking and flattening expertise of the technicians for Mohs specimens. As a limitation however, confocal images and corresponding Mohs slides were examined by the same reviewer. Notably, the 2 false negative cases were due to a highly infiltrative (sclerodermiform) BCC sample (previously discussed; Figure 5, upper panels) and a residual in situ SCC on the peripheral border (Figure 5, lower panels).

Having these limitations in mind, our data suggest that ex vivo CLSM is a fast and accurate alternative to analyze surgical margins, although with yet limited resolution (in particular for small cell clusters of highly infiltrative tumours). Currently, our data suggest that ex vivo CLSM using Histolog® Scanner V2 is mostly suited for the assessment of non‐infiltrative BCCs margins, especially when conventional histopathological analysis (prone to false negative because of limited margin assessment) is used. Further prospective studies will be needed to improve and refine the place of ex vivo CLSM in the future.

## CONFLICT OF INTEREST

None to declare.

## AUTHOR CONTRIBUTIONS

**Lorenzo Grizzetti:** Data curation, Formal analysis, Methodology, Writing – original draft, Writing – review & editing; **Francois Kuonen:** Conceptualization, Data curation, Formal analysis, Methodology, Resources, Supervision, Writing – original draft, Writing – review & editing.

## ETHICS STATEMENT

The study design was approved by the institutional review board of Lausanne University Hospital CHUV, and the local ethics committee (study 2015‐00187). Each patient enrolled provided writen informed consent.

## Data Availability

All data will be available upon request.
